# miRNA-10a-5p Alleviates Insulin Resistance and Maintains Diurnal Patterns of Triglycerides and Gut Microbiota in High-Fat Diet-Fed Mice

**DOI:** 10.1155/2020/8192187

**Published:** 2020-08-17

**Authors:** Yawei Guo, Xiaohui Zhu, Sha Zeng, Mingyi He, Xiurong Xing, Changyuan Wang

**Affiliations:** ^1^Xuanwu Hospital Capital Medical University, Beijing 100053, China; ^2^Xiangtan Central Hospital, Xiangtan 411100, China

## Abstract

miRNA-10a is rhythmically expressed and regulates genes involved in lipid and glucose metabolism. However, the effects of miRNA-10a on obesity and glucose intolerance, as well as on the diurnal pattern of expression of circadian clock genes, remain unknown. We explored the effects of miRNA-10a-5p on insulin resistance and on the diurnal patterns of serum triglycerides and gut microbiota in high-fat diet- (HFD-) fed mice. The results showed that oral administration of miRNA-10a-5p significantly prevented body weight gain and improved glucose tolerance and insulin sensitivity in HFD-fed mice. Administration of miRNA-10a-5p also maintained the diurnal rhythm of *Clock*, *Per2*, and *Cry1* expression, as well as serum glucose and triglyceride levels. Surprisingly, the diurnal oscillations of three genera of microbes, *Oscillospira*, *Ruminococcus*, and *Lachnospiraceae*, disrupted by HFD feeding, maintained by administration of miRNA-10a-5p. Moreover, a strong positive correlation was found between hepatic *Clock* expression and relative abundance of *Lachnospiraceae*, both in control mice (*r* = 0.877) and in mice administered miRNA-10a-5p (*r* = 0.853). Furthermore, we found that along with changes in *Lachnospiraceae* abundance, butyrate content in the feces maintained a diurnal rhythm after miRNA-10a-5p administration in HFD-fed mice. In conclusion, we suggest that miRNA-10a-5p may improve HFD-induced glucose intolerance and insulin resistance through the modulation of the diurnal rhythm of *Lachnospiraceae* and its metabolite butyrate. Therefore, miRNA-10a-5p may have preventative properties in subjects with metabolic disorders.

## 1. Introduction

The spiraling rates of obesity and type 2 diabetes mellitus are a worldwide health problem affecting millions of people. Overeating and reduced physical exercise are the primary reasons for these worrisome phenomena. Among the many factors proven to be involved in the development of obesity, hyperglycemia, and insulin resistance, gut microbiota plays important roles. Long-term high-fat-diet (HFD) intake can result in dysbiosis of gut microbiota and obese subjects are often characterized by low microbial diversity and impairment of microbiota composition [1–3].

Circadian rhythmicity plays critical roles in maintaining physiological homeostasis and overall health. Disruption of the circadian rhythm is often related to the onset and development of obesity, type 2 diabetes, and other metabolic syndromes [4]. Previous studies using rodent models of circadian disruptions showed that HFD feeding adversely modulated circadian signaling, suggesting a bi-directional regulation between circadian signaling and diet [5]. However, the related mechanisms need further elucidation. Recently, the gut microbiome, a vital mediator for dietary composition entrainment, was found to be involved in the maintenance of circadian rhythm [6]. Importantly, the gut microbiome exhibits diurnal rhythm and produces oscillations in its metabolites, and these diurnal variations can be disrupted by HFD intake [6, 7].

MicroRNAs (miRNAs), a class of endogenously small noncoding RNAs consisting of 19–25 nucleotides, can exert inhibitory or promoting effects on the expression of target genes posttranscriptionally. Recently, these important gene-regulatory units have been shown to modulate circadian locomotor rhythm and output pathways [8, 9]. For instance, miRNA-276a and let-7 can regulate the expression of the pacemaker gene *timeless* [10, 11], while miRNA-276b can control circadian locomotor rhythm by repressing *Beadex* expression [12]. Additionally, it has been suggested that miRNAs can modulate gene expression in their target bacteria and promote their growth, consequently shaping the gut microbiota [13]. Therefore, we speculate that miRNA-10a, which is rhythmically expressed and regulates genes involved in lipid and glucose metabolism [14], may have an effect on gut microbiota and insulin sensitivity. In this study, we found that miRNA-10a-5p alleviated HFD-induced obesity, glucose intolerance, and insulin resistance. Moreover, miRNA-10a-5p maintained diurnal patterns of triglyceride and gut microbiota that were disrupted by HFD intake.

## 2. Materials and Methods

### 2.1. Animals and Experimental Design

A total of 126 male C57BL/6J mice (8 weeks old) were purchased from Huabukang Biotech Company (Beijing, China). After an acclimatization of 1 week, all mice were randomly grouped into 3 groups (*n* = 42): Control treatment, mice fed a low-fat diet and gavaged with saline; HFD treatment, mice fed an HFD and gavaged with saline; miRNA treatment, mice fed an HFD and gavaged with miRNA-10a-5p (100 pmol/mice per day). The low-fat (10%) and high-fat diet (45%) were purchased from Research Diets (New Brunswick, NJ, USA). miRNA-10a-5p mimics were purchased from Guangzhou RiboBio, Co., Ltd (Guangzhou, China). All mice were maintained on a 12 : 12 Light/Dark cycle (Zeitgeber time (ZT) 0 = 6 : 00), in plastic cages under standard conditions and were free to access feed and water. During the experiment, body weight (BW) was recorded every two weeks. After eight weeks of treatment, six mice in each group were randomly sacrificed at 6 : 00 (Zeitgeber time 0 [ZT0], lights on), 10 : 00 (ZT4), 14 : 00 (ZT8), 18 : 00 (ZT12, lights off), 22 : 00 (ZT16), and 2 : 00 (ZT20), and 6 : 00 (ZT0) (*n* = 6). Blood samples were collected by orbital bleeding, and serum was separated and maintained at -20°C. After the abdominal cavity opened, liver and colonic digesta samples were collected and immediately frozen in liquid nitrogen. The experimental protocol was approved by the Protocol Management and Review Committee of Xuanwu Hospital Capital Medical University, and mice were treated according to the animal care guidelines of Xuanwu Hospital Capital Medical University (Beijing, China).

### 2.2. Intraperitoneal Glucose and Insulin Tolerance Test

Intraperitoneal glucose test (IGTT) and insulin tolerance test (ITT) were performed after seven weeks of treatment. Animals were intraperitoneally injected with 1.0 g glucose or 0.65 U insulin per kg BW after a six-hour fasting period. After glucose or insulin injection, blood from the tail vein was used for the measurement of glucose concentration at 0, 30, 60, and 120 min, using a One Touch Ultra Easy glucometer.

### 2.3. Gene Expression Analysis

Total RNA was extracted from the liver as previously described [15]. cDNA was synthesized using SuperScript II (Invitrogen, Shanghai, China). Primers were mixed with SYBR Green PCR mix (Invitrogen) to amplify *Clock*, *Per2*, and *Cry1* by Real-time quantitative PCR analysis. Results of gene expression were calculated using the comparative CT method normalizing target mRNA to *β*-actin. Primers are used as previously described [6] and listed in Table 1.

### 2.4. Determination of Serum Concentration of Glucose, Triglyceride, and Cholesterol

Glucose concentration was analyzed with the commercially available kit (Nanjing Jiancheng Bioengineering Institute, Nanjing, China), and triglyceride (TG) and cholesterol (Chol) contents were determined with commercially available kits (Beijing Strong Biotechnologies, Inc. Beijing, China).

### 2.5. Microbiota Profiling

Samples of colonic digesta were used for the extraction of total genome DNA and amplification was done using a specific primer with a barcode (16S V3+V4). Sequencing libraries were generated and analyzed as previously described [16]. Operational taxonomic units (OTUs) were performed for genomic prediction of microbial communities by Tax4Fun analysis as previously described [17].

### 2.6. Determination of SCFA Concentrations

After the addition of deionized precold water, samples of colonic digesta were put intermittently on a vortex mixer for 2 min. Then, the samples were maintained at 4°C for 15 min and centrifuged at 5 000 g for 15 min at 4°C. The supernatant was determined by injection onto the chromatographic system as previously described [18].

### 2.7. Bacterial Gene Quantification

Samples of colonic digesta were used for the determination of butyryl-CoA: acetate CoA-transferase (*But*) gene copy number. Genes were quantified by determining a standard curve and primers are used as previously described [6] and listed in Table 1.

### 2.8. Statistical Analysis

Statistical analyses were performed using ANOVA and followed by multiple comparisons using Bonferroni analysis (SPSS 18 software). For the time series, multivariate analysis of variance was performed by Duncan's test. Data are presented as means ± SEM. Significance was accepted at *P* < 0.05. Matrices for Pearson's r correlation coefficients were generated in GraphPad Prism (7.03) software. Rhythmicity was detected by CircWave v1.4 software (https://www.euclock.org/).

## 3. Results

### 3.1. Effects of miRNA-10a-5p on Body Weight Gain, IGTT, and ITT in HFD-Fed Mice

**Figure 1:**
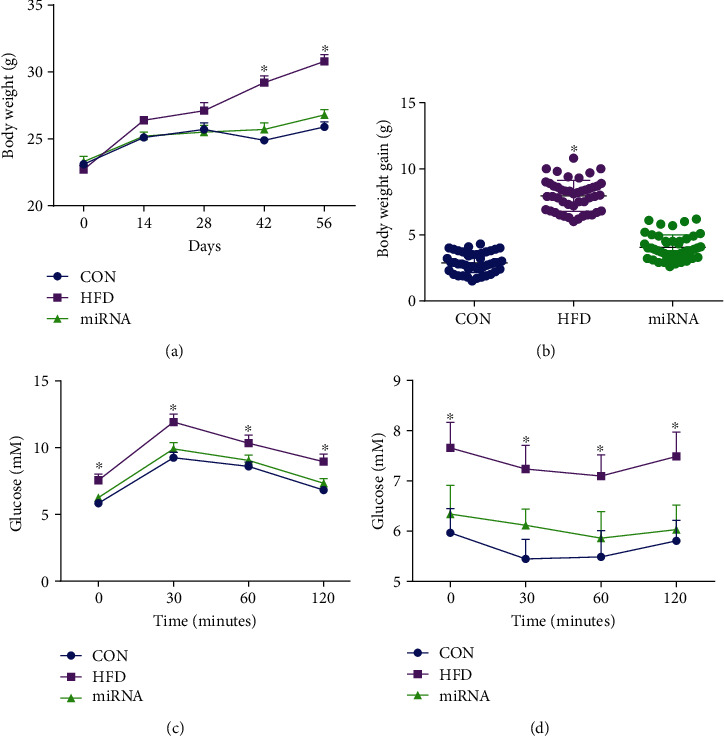
Effects of miRNA-10a-5p on body weight gain, IGTT, and ITT in high-fat diet-fed mice. (a) body weight; (b) body weight gain; (c) intraperitoneal glucose test; (d) insulin tolerance test. Data were expressed as Mean ± SEM. ^∗^*P* < 0.05.

As shown in Figure 1(a), body weight significantly increased after HFD intake for 8 weeks, while the administration of miRNA-10a-5p prevented this change. The body weight gain of control mice during the experiment was significantly lower than that of HFD-fed mice, while no significant difference in body weight was observed between control mice and HFD-fed mice administrated with miRNA-10a-5p (Figure 1(b)). The results of IGTT (Figure 1(c)) and ITT (Figure 1(d)) showed that HFD-fed mice had decreased glucose tolerance and insulin sensitivity, while miRNA-10a-5p administration improved these parameters in HFD-fed mice.

### 3.2. Effects of miRNA-10a-5p on Diurnal Rhythms of Hepatic Clock Gene and Serum Lipids in HFD-Fed Mice

**Figure 2:**
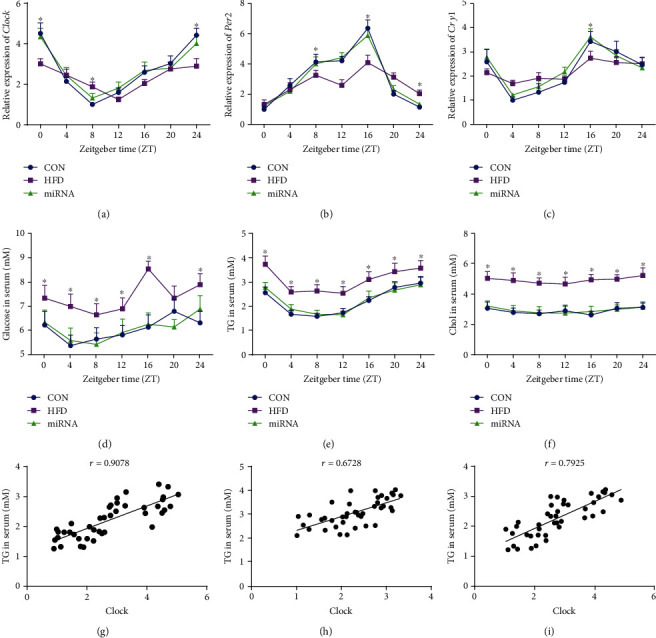
Effects of miRNA-10a-5p on diurnal rhythms of the hepatic clock gene and serum lipids in high-fat diet-fed mice. (a) Relative expression of *Clock*; (b) relative expression of *Per2*; (c) relative expression of *Cyr1*; (d) glucose content in serum; (e) triglyceride content in serum; (f) cholesterol content in serum; correlation between triglyceride content and *Clock* expression in CON group (g), HFD group (h), and miRNA group (i). Data were expressed as Mean ± SEM. ^∗^*P* < 0.05.

As shown in Figures 2(a)–2(c), the HFD altered circadian clock pattern of hepatic *Clock*, *Per2*, and *Cry1* mRNA expression as shown in previous studies [6, 7]. *Clock*, *Per2*, and *Cry1* mRNA expression in mice administrated with miRNA-10a-5p showed daily rhythms similar to those in control mice (Table 2). We found that HFD significantly increased glucose, triglycerides, and cholesterol levels in the sera of mice compared with those in the sera of mice on a low-fat diet, while there was no significant difference in these parameters between the control mice and HFD-fed mice administrated with miRNA-10a-5p (Figures 2(d)–2(f)). However, serum glucose level only showed rhythmicity in control mice, while serum triglyceride content showed rhythmicity both in control mice and HFD-fed mice administered with miRNA-10a-5p (Table 3).

To determine whether rhythmicity of serum triglycerides was related to hepatic clock gene expression, we conducted Pearson correlation analysis between triglyceride levels and circadian clock gene expressions. A strong positive correlation was observed between serum triglyceride content and hepatic *Clock* expression in control mice (*r* = 0.9078) (Figure 2(g)). Meanwhile, there was a less positive correlation between serum triglyceride levels and hepatic *Clock* expression in both control mice (*r* = 0.6728) (Figure 2(h)) and HFD-fed mice administrated with miRNA-10a-5p (*r* = 0.7925) (Figure 2(i)).

### 3.3. Effects of miRNA-10a-5p on Diurnal Rhythms of Gut Microbiota in HFD-Fed Mice

**Figure 3:**
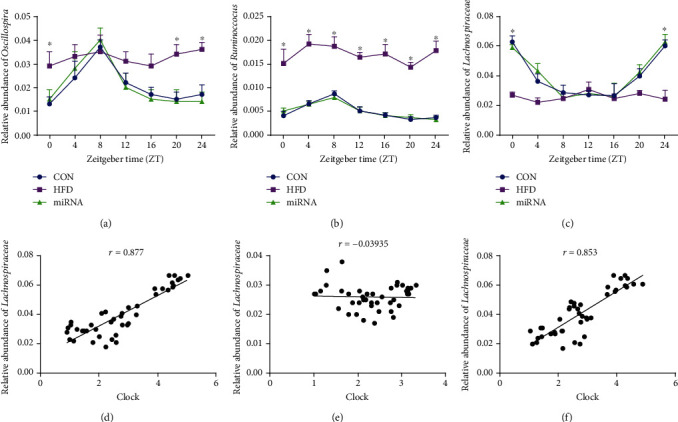
Effects of miRNA-10a-5p on diurnal rhythms of gut microbiota in high-fat diet-fed mice. (a) Relative abundance of *Oscillospira*; (b) relative abundance of *Ruminococcus*; (c) relative abundance of *Lachnospiraceae*; correlation between triglyceride content and *Clock* expression in CON group (d), HFD group (e), and miRNA group (f). TG: glycerides; Chlo: cholesterol. Data were expressed as Mean ± SEM. ^∗^*P* < 0.05.

The circadian rhythms of gut microbiota and the disturbing effects of HFD have been previously reported [6, 19]. Our results further confirmed that three genera of microbes, *Oscillospira*, *Ruminococcus*, and *Lachnospiraceae*, undergo diurnal oscillations (Table 4; Figures 3(a)–3(c)). However, as previously described [6, 7], HFD disrupted their diurnal rhythms. Importantly, HFD decreased *Lachnospiraceae* abundance at ZT0, while it increased *Ruminococcus* abundance in all the time points. The administration of miRNA-10a-5p maintained the diurnal rhythms of the aforementioned microbes.

Pearson's *r* coefficient indicated positive correlations between hepatic *Clock* expression and relative abundance of *Lachnospiraceae*, both in control mice (*r* = 0.877) (Figure 3(d)) and in HFD-fed mice administered with miRNA-10a-5p (*r* = 0.853) (Figure 3(f)). However, only a marginal negative correlation was observed between hepatic *Clock* expression and the relative abundance of *Lachnospiraceae* in HFD-fed mice (*r* = −0.03935) (Figure 3(e)).

### 3.4. Effects of miRNA-10a-5p on Diurnal Rhythms of Butyrate Content and *But* Gene Expression in HFD-Fed Mice

**Figure 4:**
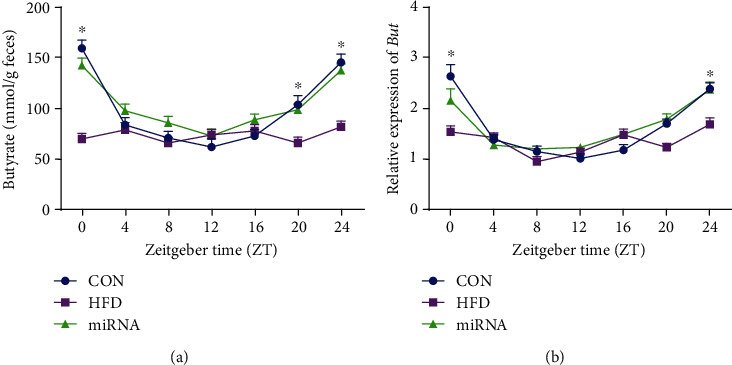
Effects of miRNA-10a-5p on diurnal rhythms of butyrate content and gene expression of *But* in high-fat diet-fed mice. (a) Relative abundance of Butyrate in feces; (b) relative expression of *But*. Data were expressed as Mean ± SEM. ^∗^*P* < 0.05.

As shown in Figure 4(a), we observed diurnal oscillation in fecal butyrate and disruption of its daily rhythm by HFD. Administration of miRNA-10a-5p maintained the diurnal rhythm of the butyrate level. Additionally, administration of miRNA-10a-5p restored the diurnal oscillation of abundant expression of the microbial gene *But*, which encodes the enzyme involved in butyrate synthesis, after the disruption caused by HFD intake (Table 4; Figure 4(b)).

## 4. Discussion

Long-term HFD intake can lead to dysfunction of glucose and lipid metabolism and dysbiosis of gut microbiota, furthering the development of metabolic diseases. Overexpression of miRNA-10a has been proven to inhibit genes involved in lipid synthesis and gluconeogenesis. We found that oral administration of miRNA-10a-5p prevented HFD-induced body weight gain and improved glucose tolerance and insulin sensitivity in mice. These results suggest that miRNA-10a-5p may have preventive effects against obesity and type 2 diabetes. Surprisingly, miRNA-10a-5p maintained the diurnal rhythms of serum triglyceride levels and expression of the hepatic clock genes *Clock*, *Per2*, and *Cry1* that were disrupted by HFD intake. Moreover, the diurnal rhythms of the bacterium *Lachnospiraceae* and its metabolite butyrate were maintained by miRNA-10a-5p administration. As expected, we found that *Lachnospiraceae* abundance showed a strong correlation with the expression of the *Clock* gene. Based on these results, we suggest that miRNA-10a-5p may alleviate HFD-induced insulin resistance through the regulation of the diurnal rhythm of *Lachnospiraceae* and its metabolite butyrate.

HFD-induced disorders of glucose and lipid metabolism are characterized by abnormally high glucose and triglyceride levels in the serum. In the present study, the decreased glucose and triglyceride levels may have resulted from the inhibitory effects of miRNA-10a-5p on hepatic expression of the genes involved in lipid synthesis and gluconeogenesis [14]. Furthermore, we confirmed that glucose and triglyceride levels in the serum of mice showed daily variations, as reported in previous studies [7, 20], and miRNA-10a-5p reestablished the triglyceride diurnal rhythm that was disrupted by HFD intake. However, the glucose diurnal rhythm was altered by HFD, in contrast to that in a previous study [7], and miRNA-10a-5p did not restore the diurnal rhythm of glucose level. We speculate that these contradictory results were because of the time of HFD feeding. Based on these results, we suggest that miRNA-10a-5p has an important effect on the maintenance of both the circadian clock pattern of triglyceride and glucose. These effects were further corroborated by the strong positive correlation between serum triglyceride levels and hepatic *Clock* expression in mice administrated with miRNA-10a-5p as well as in control mice.

It is suggested that gut microbes undergo diurnal variations [6]. Moreover, the composition and function of gut microbes can be influenced by dietary factors [6]. As expected, we found that three genera of microbes—*Oscillospira*, *Ruminococcus*, and *Lachnospiraceae*—showed diurnal rhythms. However, their diurnal oscillations were disturbed by HFD intake, in line with the results of previous studies showing that HFD disrupted the diurnal pattern of gut microbiota composition and their functions [6, 7]. MiRNAs play vital roles in the process by which the host shapes the gut microbes. Notably, miRNAs are not only critical for the homeostasis of gut microbiota in normal conditions but also provide preventative and therapeutic benefits in diseased subjects by maintaining gut microbiota. Since miRNA-10a-5p is rhythmically expressed and can regulate the expression of circadian rhythm genes, it is reasonable that miRNA-10a-5p can affect the diurnal rhythm of gut microbes. The restoration of the diurnal rhythms of *Oscillospira*, *Ruminococcus*, and *Lachnospiraceae* by the administration of miRNA-10a-5p in HFD-fed mice confirmed this theory. Moreover, the strong correlation between hepatic *Clock* expression and relative abundance of *Lachnospiraceae* in mice administrated with miRNA-10a-5p suggested that miRNA-10a-5p alleviated HFD-induced dysbiosis of gut microbiota by promoting diurnal rhythms. However, the mechanisms by which miRNA-10a-5p affects the growth of *Oscillospira*, *Ruminococcus*, and *Lachnospiraceae* have not been explored and need to be elucidated in future studies.

*Lachnospiraceae* is characterized by its ability to synthesize butyrate [21, 22]. We found that the changes in butyrate content in feces were accompanied by changes in *Lachnospiraceae* count. The beneficial effects of butyrate on the alleviation of HFD-induced metabolic disorders have been widely reported. Butyrate supplementation prevents obesity, alleviates insulin resistance, improves glucose tolerance, and decreases fat accumulation in HFD-fed mice [23–25]. Consequently, we theorize that miRNA-10a-5p alleviates insulin resistance mainly through the modulation of the diurnal rhythm of *Lachnospiraceae* and its metabolite butyrate. The effects of miRNA-10a-5p on butyrate content were further confirmed by its effects on the gene expression of *But*, which encodes the enzyme involved in butyrate production.

In conclusion, our results suggest that oral administration of miRNA-10a-5p protected against obesity, glucose intolerance, and insulin resistance in HFD-fed mice. Additionally, miRNA-10a-5p maintained the diurnal rhythms of hepatic clock genes, serum triglyceride levels, and *Lachnospiraceae* abundance in feces. Moreover, a strong positive correlation was observed between the hepatic expression of the *Clock* gene and serum triglyceride levels and *Lachnospiraceae* abundance. Along with the increase in *Lachnospiraceae* numbers, butyrate content in feces also resumed a diurnal rhythm after miRNA-10a-5p administration in HFD-fed mice. In summary, we suggest that miRNA-10a-5p may improve HFD-induced metabolic disorders through the modulation of the diurnal rhythm of *Lachnospiraceae* and its metabolite butyrate.

## Figures and Tables

**Table 1:** Primers for RT-qPCR.

Gene	5′-3′ primer sequence
*Clock*	F: ACCACAGCAACAGCAACAAC
	R: GGCTGCTGAACTGAAGGAAG
*Per2*	F: TGTGCGATGATGATTCGTGA
	R: GGTGAAGGTACGTTTGGTTTGC
*Cry1*	F: CACTGGTTCCGAAAGGGACTC
	R: CTGAAGCAAAAATCGCCACCT
*β*-Actin	F: TGTCCACCTTCCAGCAGATGT
	R: AGCTCAGTAACAGTCCGCCTAGA
*But*	F: TCAAATCMGGIGACTGGGTWGA
	R1: TCGATACCGGACATATGCCAKGAG
	R2: TCATAACCGCCCATATGCCATGAG
16 s	F: TCCTACGGGAGGCAGCAGT
	R: GGACTACCAGGGTATCTAATCCTGTT

**Table 2:** Mesor, amplitude, and acrophase of mRNA levels of clock genes in the liver of mice.

Gene	Group	Acrophase	Mesor	Amplitude	*P* value
*Clock*	CON	23.01	2.75	0.72	<0.05
	HFD				NS^∗^
	miRNA	23.21	2.77	0.50	<0.05
*Per2*	CON	13.05	3.07	0.47	<0.05
	HFD				NS
	miRNA	13.39	3.06	0.57	<0.05
*Cry1*	CON	20.64	2.22	1.19	<0.01
	HFD				NS
	miRNA	20.46	2.36	1.06	<0.05

^∗^NS: not significant.

**Table 3:** Mesor, amplitude, and acrophase of glucose and triglycerides in the serum of mice.

Item	Group	Acrophase	Mesor	Amplitude	*P* value
Glucose	CON	23.10	6.04	0.22	<0.01
	HFD				NS^∗^
	miRNA				NS
Triglycerides	CON	22.63	2.22	0.51	<0.05
	HFD				NS
	miRNA	22.90	2.27	0.42	<0.01

^∗^NS: not significant.

**Table 4:** Mesor, amplitude, and acrophase of gut microbiota, butyrate content, and *But* expression.

Item	Group	Acrophase	Mesor	Amplitude	*P* value
*Oscillospira*	CON	5.92	0.021	0.008	<0.05
	HFD				NS^∗^
	miRNA	5.72	0.021	0.011	<0.05
*Ruminococcus*	CON	5.33	0.005	0.002	<0.05
	HFD				NS
	miRNA	4.48	0.005	0.002	<0.05
*Lachnospiraceae*	CON	23.95	0.040	0.004	<0.05
	HFD				NS
	miRNA	23.97	0.041	0.002	<0.05
*Butyrate*	CON	23.73	99.00	6.35	<0.05
	HFD				NS
	miRNA	23.94	102.71	1.15	<0.05
	CON	23.74	1.62	0.09	<0.05
*But*	HFD				NS
	miRNA				NS

^∗^NS: not significant.

## Data Availability

The data used to support the findings of this study are included within the article.
